# The OPTION trial: outpatient induction of labour – study protocol for a prospective, non-inferiority, multicentre randomised controlled trial

**DOI:** 10.1136/bmjopen-2024-093972

**Published:** 2025-08-13

**Authors:** Verena Sengpiel, Heléne Sangskär, Ulla-Britt Wennerholm, Helen Elden, Kristina Gemzell-Danielsson, Sofie Graner, Tove Wallström, Susanne Hesselman, Magnus Domellöf, Maria Jonsson, Sophia Brismar Wendel, Andreas Herbst, Helena Kopp-Kallner, Ylva Carlsson

**Affiliations:** 1 Department of Obstetrics and Gynecology, University of Gothenburg Sahlgrenska Academy, Goteborg, Sweden; 2 Department of Obstetrics and Gynecology, Sahlgrenska Universitetssjukhuset Östra sjukhuset, Goteborg, Sweden; 3 Department of Obstetrics and Gynecology, Sahlgrenska University Hospital, Goteborg, Sweden; 4 Department of Obstetrics and Gynecology, Institute for Clinical Sciences, University of Gothenburg Sahlgrenska Academy, Goteborg, Sweden; 5 Institute of Clinical Sciences, Sahlgrenska Academy, University of Gothenburg, Goteborg, Sweden; 6 Health and Care Sciences, Goteborgs Universitet, Goteborg, Sweden; 7 Department of Obstetrics and Gynecology, Sahlgrenska Sjukhuset, Goteborg, Sweden; 8 Department of Women's and Children's Health, Karolinska Institute, Stockholm, Sweden; 9 WHO-centre, Gynecology and Reproductive medicine, Karolinska University Hospital, Stockholm, Sweden; 10 Maternity BB, Danderyds Sjukhus AB, Stockholm, Sweden; 11 Center for Pharmacoepidemiology, Karolinska Institute, Stockholm, Sweden; 12 Department of Clinical Science and Education, Hospital Södersjukhuset, Stockholm, Sweden; 13 Department of Obstetrics and Gynaecology, Hospital Södersjukhuset, Stockholm, Sweden; 14 Department of Women's and Children's Health, Uppsala Universitet, Uppsala, Sweden; 15 Department of Obstetrics and Gynaecology, Falun Hospital, Falun, Sweden; 16 Department of Clinical Sciences, Pediatrics, Umeå Universitet Medicinska fakulteten, Umea, Sweden; 17 Department of Clinical Sciences, Karolinska Institutet, Stockholm, Sweden; 18 Department of Obstetrics and Gynaecology, Danderyd University Hospital, Stockholm, Sweden; 19 Institution for Clinical Sciences, Lunds Universitet, Lund, Sweden; 20 Department of Obstetrics and Gynaecology, Skanes universitetssjukhus Malmo, Malmo, Sweden; 21 Department of Clinical Sciences at Danderyds Hospital, Karolinska Institutet, Stockholm, Sweden; 22 Obstetrics and Gynecology, Sahlgrenska Academy, Goteborg, Sweden; 23 Obstetrics and Gynecology, University of Gothenburg Institute of Clinical Sciences, Goteborg, Sweden

**Keywords:** Maternal medicine, NEONATOLOGY, Randomized Controlled Trial, HEALTH ECONOMICS, Patient Satisfaction, Pregnant Women

## Abstract

**Introduction:**

Sweden, as many other high-income countries, has adopted guidelines to offer induction of labour at 41+0 gestational weeks to decrease the risk for perinatal death. As more than 20% of the pregnant population reach this gestational age, and along with other contributing factors, induction rates have increased up to 30% in many countries. Both women and care providers have raised the question if outpatient induction could be a convenient, safe and economic alternative, reducing the burden on inpatient care in maternity hospitals. Before introducing outpatient induction into clinical routine, studies need to assure safety for the child and woman as well as efficacy of the method.

**Method and analysis:**

A register-based randomised controlled multicentre non-inferiority trial to study if outpatient induction in low-risk inductions is (1) as safe for the child (perinatal composite of mortality and morbidity) and (2) as effective (proportion of vaginal deliveries) as inpatient induction at the hospital. Secondary outcomes are further health outcomes, experiences of pregnant women, partners and care providers, health economics and future pregnancy outcome. Participating women with a singleton pregnancy and unripe cervix between 37+0 and 41+6 gestational weeks planned for low-risk induction will undergo induction of labour with either a balloon catheter or oral misoprostol according to clinical practice at the study site and the woman’s informed choice. Randomisation will allocate women to either outpatient (home or patient hotel) or inpatient induction (standard care). Women undergoing outpatient induction can remain at home for up to 2 days, with an assessment after 24 hours including cardiotocography. Once active labour ensues, all women will receive standard care in the hospital.

The assessment of non-inferiority will involve a two-sided 95% CI and 80% power, requiring randomisation of 8891 women to ensure a probability of at least 0.80 that the upper limit of a two-sided 95.7% CI for a difference in the primary safety outcome is below the non-inferiority margin of 1.5%. 31 of the 45 delivery units in Sweden are currently recruiting. Data will be collected from the electronic case report form and Swedish healthcare registers. Questionnaire and qualitative interview-based studies will be performed to explore experiences of pregnant women, partners and care providers. Additionally, a health economic evaluation will be performed.

**Ethics and dissemination:**

The Swedish Ethical Review Authority approved the study (3 June 2020; 2020-02675 with amendments 2021-03045, 2022-00865-02, 2023-01252-02, 2024-00560-02, 2024-2024-04597-02). The Swedish Medical Products Agency approved the study for the medication arm (25 August 2020, EudraCT number: 2020-000233-41; 5.1-2020-60240 with amendments 5.1-2022-73500, 5.1-2023-630). Due to changed regulation, in 2023, the study medication arm was transferred and approved by the European Medicines Agency (23 October 2023, EU CT Number: 2023-507164-39-00; CTIS 5.1.2-2023-099775 with amendments 5.1.2-2024-081916, 5.1.2-2025-036291). The Swedish Medical Products Agency approved the study for the medical device arm (6 April 2021, CIV-ID: CIV-20-09-034712; 5.1-2021-14812 with amendments 5.1-2022-14252, 5.1-2023-596, 5.1-2024-8886, 5.1-2024-55554). The medical device arm was transferred to Regulation (EU) 2017/745 (23 December 2024, 5.1-2025-24242 and amendment 5.1-2025-6050). The study will involve more than 80% of all delivery units in Sweden, which will allow for a smooth implementation of any new routine after the study’s conclusion. Results will be published in relevant scientific journals, presented at national and international conferences, and communicated to participants and relevant institutions through the Outpatient Induction study homepage (www.optionstudien.se), the webinars of the Swedish Network for National Clinical Studies in Obstetrics and Gynecology (www.snaks.se) as well as social and public media.

**Trial registration number:**

EudraCT No: 2020-000233-41, after transfer to the European Medicines Agency EU CT Number: 2023-507164-39-00; CIV-ID 20-09-034712.

**Strengths and limitations of this study**

- The study has the statistical power to assess the safety and efficacy of mechanical and pharmacological labour induction at home but lacks the statistical power to assess the safety of each method independently.
- In the Outpatient Induction trial, participants are randomised to outpatient or inpatient induction of labour in a controlled trial setting with stratification for relevant factors influencing the outcome.
- After initiation of induction, women are observed for 45 min before randomisation to ensure eligibility and minimise differences between the intention-to-treat and per-protocol populations.
- The results will be applicable to women with an expected low risk of complications during the ripening phase with a singleton pregnancy and unripe cervix between 37+0 and 41+6 gestational weeks, for example, excluding women with a previous caesarean section.
- The results regarding patient experience will mainly apply to women open to outpatient induction since women with major concerns about outpatient induction are likely to decline participation.

## Introduction

 Induction of labour (IOL) rates are increasing globally especially since WHO recommended IOL for women with low-risk pregnancies at 41+0 gestational weeks.^1^ After publication of the SWEdish Post-term Induction Study, the SWEPIS trial^2^ and the meta-analysis by Alkmark *et al*,^3^ Sweden amended guidelines offering IOL at 41 instead of 42 gestational weeks. Today, in most high-income settings, about 30% of all births are induced,^4^ currently 28% in Sweden.^5^ When labour starts spontaneously, women usually stay at home during the latent phase and are admitted to the hospital on entering active labour. How labour is best induced depends on the status of the cervix. When the cervix is ripe, labour can be induced by artificial rupture of the membranes. Otherwise, cervical ripening is achieved by either inserting a balloon catheter into the cervix or using prostaglandins.^6 7^

Both pregnant women and care providers have raised the question if outpatient IOL could be a convenient, safe and economic alternative, and outpatient IOL is already implemented into clinical routine in some parts of the world.^8 9^ To assess the need for further knowledge, we performed a systematic review and meta-analysis.^10 11^ Few studies had investigated if IOL by cervical ripening could be performed in an outpatient setting. None of the studies were powered to study safety aspects of rare outcomes such as perinatal or maternal death or severe morbidity. Nor were the studies sufficiently large or homogenous to allow for a meta-analysis of efficacy—defined as proportion of vaginal deliveries. A Cochrane report from 2020 came to similar conclusions.^12^

There are few studies available on women’s experiences of outpatient IOL. Studies have indicated that the home environment contributes to physical and emotional comfort and might enhance women’s birthing experiences.^13^,^18^

Moreover, the partner’s experience has hardly been studied,^19^ despite the partner often being the main support person during outpatient IOL. Only a handful of studies have investigated healthcare provider experience, yielding both positive feedback and certain concerns.^20 21^

In addition to medical outcomes and patient experience, health economics plays a significant role in the consideration of introducing new care settings. As previous studies have not been adequately powered to evaluate the most severe safety outcomes, health economic evaluations have been unable to accurately assess the costs associated with outpatient IOL.^22^,^24^

The objective of the Outpatient Induction (OPTION) multicentre register-based randomised controlled trial (R-RCT) is to evaluate if outpatient IOL in low-risk IOLs is non-inferior to inpatient IOL regarding safety for the child (as measured by a composite outcome for peri-neonatal morbidity and mortality) and efficacy (determined by the proportion of vaginal delivery). Additionally, pregnancy outcomes, including maternal safety variables, the acceptability and experience of the woman, her partner and the care providers, health economic aspects as well as future pregnancy outcomes will be studied.

## Methods and analysis

This protocol was written in accordance with the “Standard Protocol Items: Recommendations for Interventional Trials (SPIRIT)”^25^ guidelines for randomised controlled trial (RCT) protocols.

### Design, setting and study population

**Figure 1:**
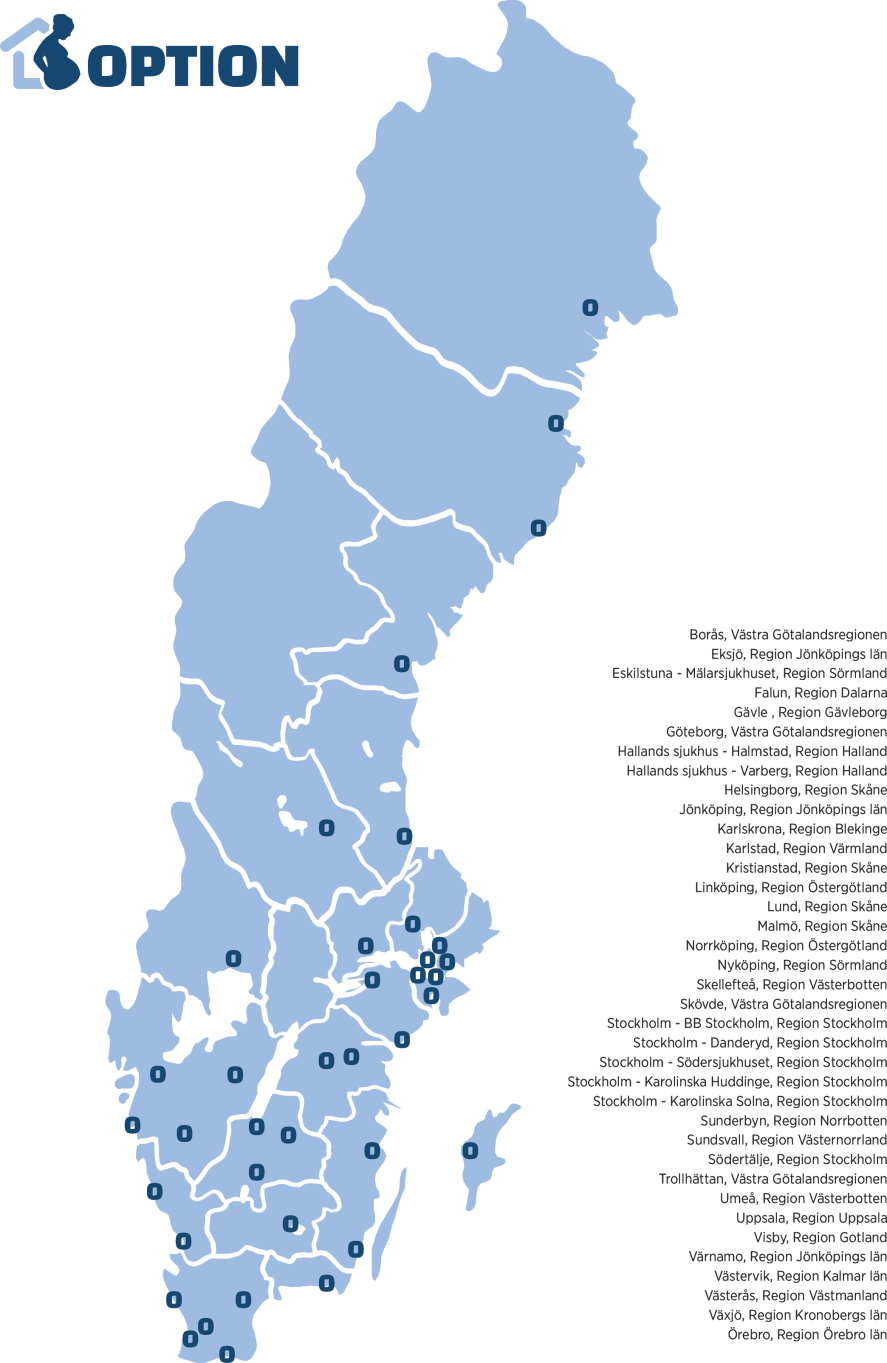
Participating maternity hospitals and counties in Sweden.

OPTION is an R-RCT with randomisation by a module linked to the Swedish Pregnancy Register (SPR).^26^ Out of 45 Swedish maternity hospitals, 37 hospitals representing about 91 000 out of approximately 101 000 deliveries per year are planning to participate or are already recruiting (n=31), covering both rural and urban areas as well as small and large hospitals (figure 1). Women aged 18 to 45 years planned for IOL between 37+0 and 41+6 gestational weeks will be screened for eligibility (table 1). Recruitment started on 1 December 2021 and until 10 July 2025, a total of 2172 women have been randomised. Recruitment is planned to be completed by 31 December 2028.

**Table 1:** Inclusion and exclusion criteria in the OPTION trial

Inclusion criteria	Exclusion criteria
Based on medical history	
Women 18–45 years old Able to communicate with the hospital Uncomplicated live singleton pregnancy Pregnancy week ≥37+0 to 41+6 according to crown rump length (CRL) or biparietal diameter (BPD <55 mm) at first or second trimester ultrasound Stable cephalic presentation Able to reach the hospital in a reasonable time, at the discretion of the investigator with a maximum of 60 min as a benchmark^54^	Previous uterine surgery with uterine scar, for example, caesarean section or myomectomy Pregestational (diagnosis before pregnancy or before gestational week 25) or insulin-treated gestational diabetes Dietary or metformin-treated gestational diabetes with small or large for gestational age fetus Pre-eclampsia or instable hypertensive disease Intrahepatic cholestasis with serum bile acids ≥40 µmol/L Intrauterine foetal death (IUFD) in previous pregnancy Knownsmall for gestational age (SGA) defined as <2 SD according to Marsal*et al*^55^or intrauterine growth restriction (IUGR) Known foetal malformations or other foetal condition affecting the delivery or immediate care of the newborn Congenital uterine malformation which may affect safety Other condition requiring inpatient care, for example, delivery within 60 min from arriving at the hospital in previous pregnancy Known allergy to any of the components of the balloon catheter or oral misoprostol (for the respective method only)
Based on clinical examination before start of induction including Leopold’s manoeuvres, digital cervical exam, abdominal ultrasound, blood pressure and CTG scan, if indicated temperature	
Stable cephalic presentation with Bishop score <6 CTG classified as normal according to the antepartal Swedish Society of Obstetrics and Gynaecology (Svensk Förening för Obstetrik och Gynekologi, SFOG) criteria (www.ctgutbildning.se)	Small for gestational age (SGA) defined as <2 SD and in case of diabetes either large for gestational age (LGA) defined as >2 SD according to Marsal *et al*^55^ or suspected growth acceleration (according to the hospital’s guidelines). If the criteria outlined below are not met in their respective situation, the patient shall be excluded from the study: In case of late term ≥41+0 to 41+6 weeks, abdominal ultrasound will be performed and mean abdominal diameter (MAD) must be ≥110 mm. In case MAD <110 mm, the foetal weight will be estimated to exclude SGA fetus and if SGA <2 SD according to Marsal *et al*^55^, the patient should be excluded In case of dietary or metformin-treated gestational diabetes, foetal weight needs to be or have been estimated by abdominal ultrasound within the last 3 weeks before induction and the result needs to be within normal range In case of stable hypertension, foetal weight needs to be estimated by abdominal ultrasound within the last 2 weeks before induction and showing no SGA/IUGR fetus In case of indication for induction being prolonged latent phase, maternal age, mild intrahepatic cholestasis, pelvic girdle pain, PROM or psychosocial, fundal height measurement according to the Swedish reference curves must be normal. In case of not-normal fundal height measurement, foetal weight estimation must be performed and showing no SGA/IUGR For all other indications, foetal weight is estimated by abdominal ultrasound at the discretion of the investigator. Oligohydramniosis: deepest vertical pocket <20 mm or amniotic fluid index <50 mm Polyhydramniosis: amniotic fluid index >250 mm or single deepest pocket >80 mm Maternal pyrexia >38°C Known low-lying placenta (less than 20 mm from internal os measured by ultrasound) Regarding premature rupture of membranes (PROM): PROM is exclusion criteria for catheter method PROM is exclusion criteria for misoprostol if: Time point for confirmed PROM with timepoint for PROM as indicated by the woman >48 hours Known colonisation with group B streptococci or previous pregnancy complication linked to group B streptococci^48^
Based on observation the first 45 min after start of induction	
In case of induction with catheter method: CTG classified as normal according to the antepartal Swedish Society of Obstetrics and Gynaecology (Svensk Förening för Obstetrik och Gynekologi, SFOG) criteria (www.ctgutbildning.se)	Any adverse events within the first 45 min after start of induction, for example, heavy bleeding, pain, PROM in case PROM was not an indication for induction of labour Start of contractions

CTG, cardiotocography; OPTION, Outpatient Induction.

#### Recruitment, screening and inclusion

Women receive written trial information through brochures and posters at antenatal care units and delivery units, as well as oral and written information when planning for IOL. Additionally, information is disseminated through public media, posts on social media platforms and the study website.^27^

**Figure 2:**
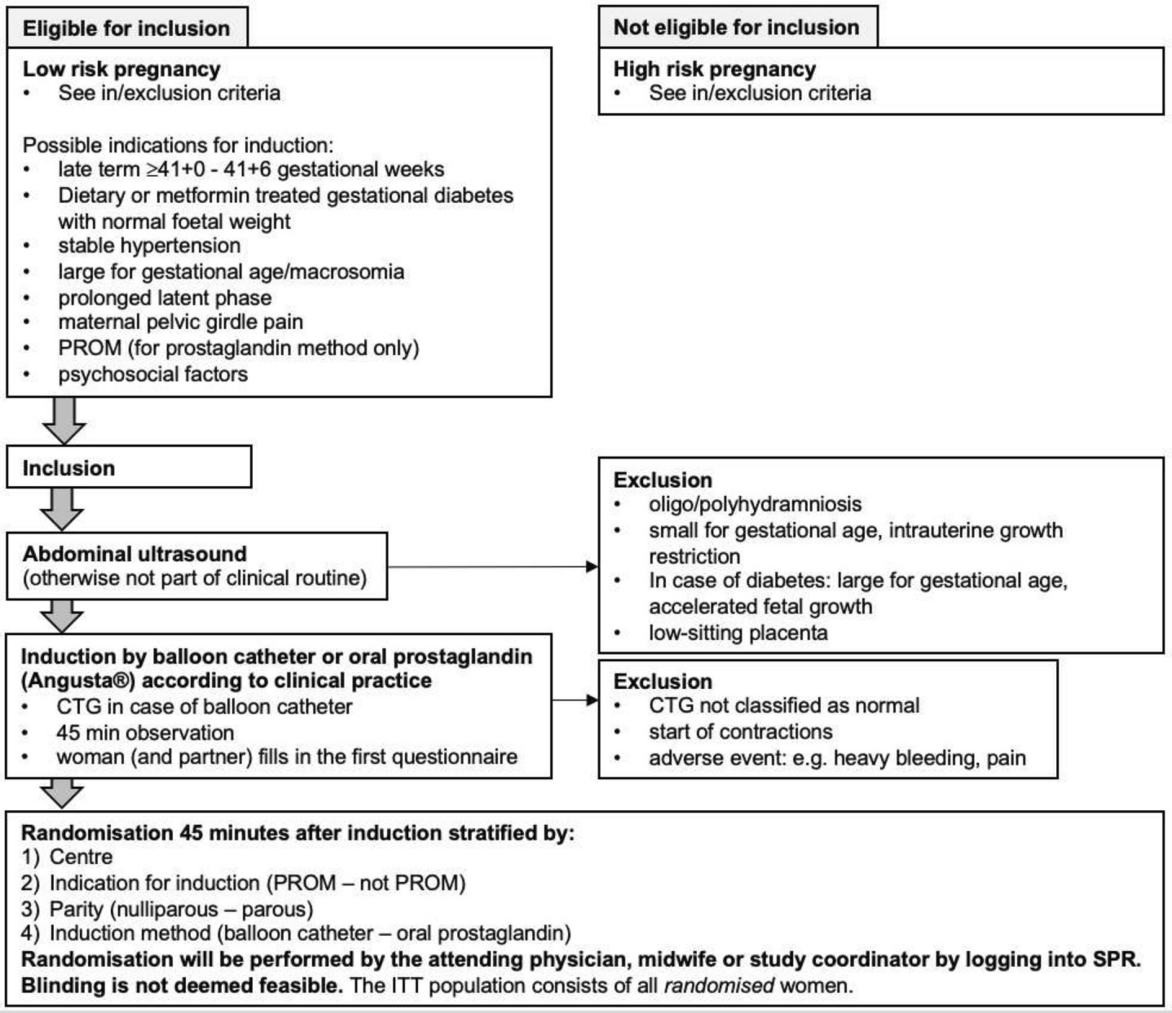
Overview over the recruitment, induction and randomisation procedure in the OPTION trial. CTG, cardiotocography; ITT, intention to treat; OPTION, Outpatient Induction; PROM, prelabour rupture of the membranes; SPR, Swedish Pregnancy Register.

Screening for eligibility takes place at the hospital visit for start of IOL including medical history, blood pressure, vaginal examination, abdominal ultrasound and cardiotocography (CTG). Women with a term, singleton, live fetus in stable cephalic presentation, unripe cervix, normal CTG prior to IOL and an anticipated uncomplicated cervical ripening phase—ie, not requiring maternal or foetal monitoring—are defined as low-risk IOL and eligible for inclusion. Detailed inclusion and exclusion criteria are presented in table 1. Partners become eligible for inclusion in the partner parts of the study if the woman is included. Women and partners may be included after oral and written informed consent is obtained, please see online supplemental material 1. For the process of recruitment, inclusion, IOL and randomisation, see even figure 2.

#### Induction of labour

IOL is initiated in the hospital and performed using methods applied in clinical routine: mechanical methods—double balloon catheter (Cook Cervical Ripening Balloon with Stylet, Cook Medical, O’ Halloran Road, National Technology Park Limerick, Ireland) or single balloon catheter (see online supplemental table 1 for approved models marked as study product)—or the medical method (oral tablets of 25 µg misoprostol; Angusta, Norgine BV, Amsterdam, Netherlands). The method of IOL is chosen depending on the woman’s informed choice, local practice and the physician’s advice, and following the guidelines from the Swedish Society of Obstetrics and Gynaecology (SFOG).^28^ However, in the case of prelabour rupture of the membranes (PROM), misoprostol is the only choice. The double balloon catheter (Cook) is administered as specified by the manufacturer. Similarly, the single balloon catheter is placed by speculum/amnioscope or digitally; whereafter, the balloon is filled with up to 50 mL saline solution. A dose of 25 µg oral misoprostol is taken no closer than 2 hours apart with a maximum of eight doses per 24 hours as specified by the manufacturer.

#### Randomisation

After the balloon catheter has been placed or the woman has taken the first dose of oral misoprostol, the woman is observed for 45 min. For women who received a balloon catheter, a CTG is registered after insertion, before randomisation, see figure 2. For women who received the first dose of oral misoprostol, an additional CTG is only registered if indicated, for example, start of contractions. For exclusion criteria after initiation of IOL, see table 1. Following inclusion, but before randomisation, participating women and partners complete the first electronic questionnaire.

Randomisation is conducted online via a module linked to the SPR, accessible through a computer using a personal staff identification card and two-factor authentication. The module includes both the randomisation module and the electronic case report form (eCRF). Randomisation is performed by optimal allocation (minimisation) per study site considering (1) indication for IOL (PROM – no PROM), (2) parity (nulliparous – parous) and (3) method of IOL (balloon catheter – oral misoprostol). At study completion, allocation is 1:1 with maximum difference of two patients between the two randomised groups within each centre. Blinding is not deemed feasible for neither patient, partner, care provider, nor clinical research staff. The approach to not randomise until after an initial observational period ensures that the choice of IOL method and subsequent monitoring is not influenced by known group allocation and hence, minimises difference in intention to treat (ITT) and per-protocol populations.

#### Procedures and follow-up

The trial intervention is IOL outside of a hospital setting. For IOL due to PROM, the method of cervical ripening and whether the setting will be outpatient or inpatient is set for the first 24 hours. For IOL due to other indications, these details are specified for the first 48 hours. Thereafter, all participants are treated according to clinical routine at their hospital. Usually, IOL will proceed with oxytocin infusion in case of PROM and amniotomy and subsequent oxytocin infusion in women with intact membranes.^29^

Women randomised to inpatient IOL are monitored according to local clinical routine. Women randomised to outpatient IOL return home or to a patient hotel defined as a facility without access to medical surveillance. Participants receive oral and written information on how to proceed with IOL, when and whom to call and when to return to the hospital. Women have access to a 24/7 telephone line staffed by qualified midwives based at the maternity hospital. Women randomised to outpatient IOL receive detailed oral and written instructions to contact the hospital (and refrain from further intake of oral misoprostol) at start of contractions, in case of PROM (if PROM was not the indication for IOL), when the balloon catheter is expelled, sudden change/decrease in foetal movements, vaginal bleeding, abdominal pain, fever and/or feeling unwell, and in case of IOL due to PROM: any change in amniotic fluid colour. These women are counselled to come to the hospital for a clinical evaluation. Women are also encouraged to call in case they feel unsure about anything.

If labour does not start and no complication occurs, women in the outpatient group return for assessment after 24 hours (12 hours in case of IOL with a double balloon (Cook) catheter due to conformité européenne (CE) marking for 12 hours use only) for clinical assessment including vaginal examination of the cervix, blood pressure and CTG. Women induced due to PROM are admitted to the hospital at the latest 24 hours after start of IOL. Women induced due to other indications and a normal assessment may return home/to the patient hotel for continued cervical ripening for another 24 hours if needed.

Women in the outpatient group are admitted to the hospital if any of the following occurs after randomisation: PROM, onset of labour, complications requiring inpatient observation of the woman or fetus, the woman’s request, or when the cervical status allows for amniotomy followed by oxytocin infusion for continued IOL. On admission, remaining tablets, or expelled balloon catheters, are collected and inspected by the care providers. All births are planned to take place at the hospital.

### Outcomes

#### Primary outcomes

Two primary outcomes have been defined: (1) child safety and (2) efficacy defined as proportion of vaginal deliveries. The primary safety outcome is defined as a composite variable for perinatal mortality and morbidity including stillbirth after randomisation, neonatal death within 28 days (not including lethal malformations), Apgar score <4 at 5 min, umbilical artery pH <7.00 or base deficit >15 mmol/L, hypoxic ischaemic encephalopathy grades 1 to 3, intracranial haemorrhage, neonatal seizures, therapeutic hypothermia, meconium aspiration syndrome, mechanical ventilation (tracheal intubation) within the first 72 hours, neonatal pneumonia, neonatal sepsis or admission to the neonatal intensive care unit >48 hours duration.

#### Secondary outcomes

Secondary outcomes are further health outcomes, experiences of pregnant women, partners and care providers, health economics and future pregnancy outcome.

#### Further medical outcomes

Secondary and exploratory clinical outcomes are specified according to the recommendations in the latest Cochrane report on outpatient IOL,^30^ the CROWN (core outcomes in women’s health) initiative core outcome set for IOL^31^ as far as possible with regard to the register-based manner of data retrieval, see tables2  3.

**Table 2:** Primary health outcome variables in the OPTION trial

Variable	Type	Description	Source
**Primary outcome 1 is a composite variable for severe child morbidity and mortality including any of the following:**			
Stillbirth defined as intrauterine foetal death of a fetus that was alive at time of randomisation	Dichotomous (yes, no)	Tick box, ICD-10 O36.4	SPR
Neonatal death of a live born child that dies days 0–27, not including accidents or lethal malformation not known before randomisation	Dichotomous (yes, no)	SNQ 2101, 2103	SNQ, SCB, the Cause of Death Register
Apgar score <4 at 5 min	Dichotomous (yes, no)	SNQ 421, SPR “Apgar 5”	SNQ, SPR
pH <7.00 or base deficit >15 mmol/L in the umbilical artery	Dichotomous (yes, no)		SNQ, SPR
Hypoxic ischaemic encephalopathy I–III	Dichotomous (yes, no)	ICD-10 P91.6 or P91.0 or tick box	SNQ
Intracranial haemorrhage	Dichotomous (yes, no)	ICD-10 P10, P52	SNQ
Neonatal convulsions	Dichotomous (yes, no)	ICD-10 P90	SNQ
Therapeutic hypothermia	Dichotomous (yes, no)	Procedure code DV034	SPR, SNQ
Meconium aspiration syndrome	Dichotomous (yes, no)	ICD-10 P24.0	SNQ, SPR
Mechanical ventilation within first 72 hours	Dichotomous (yes, no)	Procedure code DG021, DG022, DG002	SNQ
Neonatal pneumonia	Dichotomous (yes, no)	ICD-10 P23	SNQ
Neonatal sepsis	Dichotomous (yes, no)	ICD-10 P36	SNQ
NICU admission >48 hours duration	Dichotomous (yes, no)		SNQ
**Primary outcome 2 is the efficacy variable defined as proportion of vaginal birth in the two groups**			
Caesarean section	Dichotomous (yes, no)	Tick box ICD-10 O82 Procedure code MCA00, MCA10, MCA20, MCA30, MCA33, MCA96	SPR
Vaginal delivery (spontaneous and instrumental)	Dichotomous (yes, no)	Tick box Spontaneous vaginal ICD-10 O80 Instrumental vaginal ICD-10 O81 Procedure code MAF00, MAF10, MAF96, MAC23 MAE00, MAE03, MAE20, MAE96	SPR

ICD-10, International Statistical Classification of Disease and Related Health Problems 10th revision; NICU, neonatal intensive care unit; OPTION, Outpatient Induction; SCB, Statistics Sweden; SNQ, the Swedish Neonatal Quality Register; SPR, the Swedish Pregnancy Register.

**Table 3:** Secondary and exploratory health outcome variables in the OPTION trial

Variable	Type	Description	Source
Secondary and exploratory outcome variables*			
The different variables being part of the primary safety composite outcome will even be studied individually in the form of exploratory analyses.			
Stillbirth defined as intrauterine foetal death of a fetus that was alive at time of randomisation	Dichotomous (yes, no)	ICD-10 O36.4	SPR
Neonatal death of a live born child that dies days 0–27, not including accidents	Dichotomous (yes, no)	SNQ 2101, 2103	SNQ, SCB, the Cause of Death Register
Apgar score <4 at 5 min	Dichotomous (yes, no)	SNQ 421, SPR “Apgar5”	SNQ, SPR
pH <7.00 or base deficit >15 mmol/L in the umbilical artery	Dichotomous (yes, no)		SNQ, SPR
Severe birth asphyxia	Dichotomous (yes, no)	ICD-10 P21.0	SNQ, SPR
Hypoxic ischaemic encephalopathy II–III	Dichotomous (yes, no)	ICD-10 P91.6B or C or tick box	SNQ
Intracranial haemorrhage	Dichotomous (yes, no)	ICD-10 P10, P52	SNQ
Neonatal convulsions	Dichotomous (yes, no)	ICD-10 P90	SNQ
Therapeutic hypothermia	Dichotomous (yes, no)	ICD-10 P80.8, P80.9, DV034	SPR, SNQ
Meconium aspiration syndrome	Dichotomous (yes, no)	ICD-10 P24.0	SNQ, SPR
Mechanical ventilation within first 72 hours	Dichotomous (yes, no)	Procedure code DG021, DG022, DG002	SNQ
Neonatal pneumonia	Dichotomous (yes, no)	ICD-10 P23	SNQ
Neonatal sepsis	Dichotomous (yes, no)	ICD-10 P36	SNQ
NICU admission >48 hours	Dichotomous (yes, no)		SNQ
Further outcomes for the child			
Obstetric brachial plexus injury	Dichotomous (yes, no)	ICD-10 P14.0, P14.1, P14.3, P14.8, P14.9	SNQ, SPR
Admission to the NICU	Dichotomous (yes, no)		SNQ
Time at NICU	Continuous (days and hours)		SNQ
Treatment for hypoglycaemia	Dichotomous (yes, no)	ICD-10 P70.3 P70.4A-B, P70.8, P70.9	SNQ, SPR
Readmission after delivery due to the child’s health until day 27 after delivery	Dichotomous (yes, no)		SNQ
Healthy person accompanying sick person (mother stays at the hospital due to need of care for the newborn) until day 27 after delivery	Dichotomous (yes, no)	ICD-10 Z76.3	SPR
Apgar score <7 at 5 min	Dichotomous (yes, no)	SNQ 241, SPR “Apgar5”	SNQ, SPR
Further outcomes for the mother within 12 weeks from delivery if not specified otherwise			
Vaginal delivery (spontaneous vs instrumental)	Dichotomous (yes, no)	Tick box Spontaneous vaginal ICD-10 O80 Instrumental vaginal ICD-10 O81 Procedure code MAF00, MAF10, MAF96, MAC23 MAE00, MAE03, MAE20, MAE96	SPR
Maternal death until 42 days after delivery that can be connected to the pregnancy	Dichotomous (yes, no)	ICD-10 O95, O97	SPR, SCB, the Cause of Death Register
Maternal death after 42 days after delivery that can be connected to the pregnancy	Dichotomous (yes, no)	ICD-10 O96	SPR, SCB, the Cause of Death Register
Pre-eclampsia	Dichotomous (yes, no)	ICD-10 O14, O15,	SPR
Gestational (pregnancy-induced) hypertension without significant proteinuria	Dichotomous (yes, no)	ICD-10 O13, O16	SPR
Precipitate labour	Dichotomous (yes, no)	ICD-10 O62.3	SPR
Hypertonic, incoordinate and prolonged uterine contractions	Dichotomous (yes, no)	ICD-10 O62.4	SPR
Uterine rupture	Dichotomous (yes, no)	ICD-10 O71.0, O71.1, Procedure code MCC00	SPR
Hysterectomy in connection with delivery	Dichotomous (yes, no)	ICD-10 O82.2, Procedure code MCA33	SPR
Cardiac arrest	Dichotomous (yes, no)	ICD-10 I46	SPR, the National Patient Register
Obstetric shock	Dichotomous (yes, no)	ICD-10 O75.1	SPR
Other severe maternal morbidity defined as admission to intensive care unit	Dichotomous (yes, no)	ICD-10 ZV049	SPR, hospital charts, the National Patient Register
Thrombosis, pulmonary embolism	Dichotomous (yes, no)	ICD-10 O22.3, O87.1, O87.3, I82.2, I82.8, I82.9, I26	SPR, the National Patient Register, Prescribed Drug Register
Obstetric embolism	Dichotomous (yes, no)	ICD-10 O88	SPR, Prescribed Drug Register
Umbilical cord prolapse	Dichotomous (yes, no)	ICD-10 O69.0, P02.4	SPR
Vaginal delivery within 24 hours (VD24)	Dichotomous (yes, no)	As above within 24 hours after start of induction	SPR
Vaginal delivery within 48 hours (VD48)	Dichotomous (yes, no)	As above within 24 hours after start of induction	SPR
Stroke	Dichotomous (yes, no)	ICD-10 I61.X, I63.X	SPR, the National Patient Register
Emergency or crash caesarean section	Dichotomous (yes, no)	ICD-10 O82.1	SPR
Indication for instrumental vaginal delivery or delivery by caesarean section		Foetal distress ICD-10 O.68.9, O36.3, Infection ICD-10 O75.3, O98.8, O98.9, Failure to progress ICD-10 O62.0-2, O62.8-9, Maternal distress during labour and delivery ICD-10 O75.0	SPR
Shoulder dystocia	Dichotomous (yes, no)	ICD-10 O66.0	SPR
Labour dystocia	Dichotomous (yes, no)	ICD-10 O.62.0-1, O62.8-9	SPR
Use of oxytocin	Dichotomous (yes, no)	DT036, DT037	SPR
Hypertonic, incoordinate and prolonged uterine contractions	Dichotomous (yes, no)	ICD-10 O62.4	SPR
Heavy vaginal bleeding before or during delivery	Dichotomous (yes, no)	ICD-10 O46, O67	SPR
Placental abruption	Dichotomous (yes, no)	ICD-10 O45	SPR
Number and reasons of visits and phone calls to the hospital in the outpatient group (balloon catheter expulsion, planned visit after 24 hours, PROM, pain, vaginal bleeding, contractions, impaired urination, foetal movements, delivery before reaching the hospital, other)			eCRF, SPR, the National Patient Register and regional registries as for example, VEGA (Vårddatabas för Enskilda patienter, Grupper och Analyser) in the Western Healthcare region
Need of additional induction method	Dichotomous (yes, no)	ICD-10 O61.X	eCRF, SPR
Infection (before, during, after delivery)	Dichotomous (yes, no)	ICD-10 O75.3, O85, O86, O91, O98, and see below	SPR, the National Patient Register, Prescribed Drug Register
Chorioamnionitis	Dichotomous (yes, no)	ICD-10 O41.1, SNQ 303	SPR, SNQ,
Urinary tract infection	Dichotomous (yes, no)	ICD-10 O86.2	SPR, the National Patient Register, Prescribed Drug Register
Endometritis	Dichotomous (yes, no)	ICD-10 O85.9, O86.1 O86.3, O86.8	SPR, the National Patient Register, Prescribed Drug Register
Wound infection	Dichotomous (yes, no)	ICD-10 O86.0	SPR, the National Patient Register, Prescribed Drug Register
Sepsis	Dichotomous (yes, no)	ICD-10 A41	SPR, the National Patient Register, Prescribed Drug Register
Fever during delivery	Dichotomous (yes, no)	ICD-10 O75.2	SPR
Fever postpartum	Dichotomous (yes, no)	ICD-10 O86.4	SPR
Need and method of pain relief during delivery		ICD-10 ZXH50, ZXH40, ZXH10, SN999	SPR
Episiotomy	Dichotomous (yes, no)	Tick box (left, median, right) Procedure code TMA00	SPR
Grades 3 or 4 perineal laceration	Dichotomous (yes, no)	Tick box sphincter, rectum ICD-10 O70.2, O70.3, MBC33	SPR
Perineal laceration	Dichotomous (yes, no)	Tick box, ICD-10 O70	SPR
Amount of postpartum bleeding	Continuous (mL)		SPR
Postpartum bleeding >1000 mL	Dichotomous (yes, no)	ICD-10 O72	SPR
Transfusion	Dichotomous (yes, no)	Procedure code DR029, DR030, DR036-039	SPR
Breastfeeding at discharge from hospital	Dichotomous (yes, no)		SPR
Breastfeeding at follow-up visit to the midwife at 8–12 weeks postpartum	Dichotomous (yes, no)		SPR
Readmission after delivery due to the mother’s health	Dichotomous (yes, no)		SPR, the National Patient Register
Experience of delivery	Continuous 1–10 Categorical		SPR
Postnatal depression	Dichotomous (yes, no)	ICD-10 F53.X	SPR
Time variables			
Time from start of induction to second stage of delivery	Continuous (hours, minutes)		SPR
Time from start of induction to delivery	Continuous (hours, minutes)		SPR
Duration of stay at the hospital	Continuous (hours, minutes)		SPR
Duration of stay at the hospital before delivery	Continuous (hours, minutes)		SPR
Duration of stay at the hospital after delivery	Continuous (hours, minutes)		SPR
Descriptive outcomes for the outpatient group only			
Delivery within 30 and 60 min from admission to hospital	Dichotomous (yes, no)		SPR
Caesarean section within 60 min from admission to hospital	Dichotomous (yes, no)		SPR
Arrival at the hospital by ambulance	Dichotomous (yes, no)		Ambureg
Delivery before reaching the hospital	Dichotomous (yes, no)		SPR

* The final statistical analysis plan will be set prior to data lock and define which variables will be studied as secondary vs exploratory variables.

Ambureg, the Swedish Ambulance Register; ICD-10, International Statistical Classification of Disease and Related Health Problems, 10th revision; OPTION, Outpatient Induction; PROM, prelabour rupture of the membranes; SCB, Statistics Sweden; SNQ, the Swedish Neonatal Quality Register; SPR, the Swedish Pregnancy Register.

#### Acceptability and experience of outpatient IOL

Further, outcomes relating to the acceptability and experience of the woman, her partner and the care providers will be studied:

Questionnaires measuring general self-efficacy (GSE),^32^ health-related quality of life (HRQL) (EuroQol Visual Analogue Scale (EQ-VAS) and EuroQol 5-Dimension (EQ-5D)),^33 34^ sense of coherence (SOC-13),^35^ pain catastrophising (PCS)^36^ and depression (The Edinburgh Postnatal Depression Scale, EPDS)^37^ at baseline are sent out by email and/or short message service (SMS) to women and partners after inclusion, but before randomisation. Three months after delivery, the GSE, EQ-VAS, EQ-5D, SOC, PCS, EPDS, childbirth experience questionnaire (CEQ2),^38^ father for the first-time questionnaire (FTFQ)^39^ and Breastfeeding Self-Efficacy Scale Short Form (BES-SF)^40^ are distributed by email and/or SMS. Furthermore, the experience of IOL will be evaluated by a questionnaire adapted from Bollapragada *et al*,^41^ allowing participants to provide free-text responses. Partners at one centre are invited to a web-based qualitative questionnaire adapted from Daniels *et al*^42^ 3 months after delivery. In all participating partners, background characteristics (age, education level, number of children and number of experienced childbirths and IOL) are registered. All questionnaires are validated in Swedish and English.

In addition to the questionnaires, a qualitative approach will involve interviewing 15–20 women and 15–20 partners 3–6 months after delivery. These informants will be strategically selected from diverse age groups, parity and socioeconomic backgrounds to ensure a comprehensive spectrum of experiences of the phenomenon (outpatient) IOL. Care providers consisting of 15–20 individuals will also be strategically selected based on various criteria including age, gender, profession (midwife, physician, assistant nurse), workplace and specific tasks, such as answering phone calls, working at the induction unit, delivery unit or postnatal care unit. Focus group or individual interviews will be conducted in Swedish or English at the hospital, in the woman’s/partner’s/healthcare provider’s home or via digital meeting options, depending on their preference. An open-ended question will be used: “Please tell me of your experience of (outpatient) induction”. Follow-up questions, such as “How did that feel” and “Can you please tell me more,” will also be asked to deepen understanding. The interviewer will create an open climate to enable the informant to find the right words to express her/his experiences.^43^ Interviews will be audiotaped and transcribed verbatim.

#### Health economic outcome

The following variables will be included in the health economic analyses: pregnancy, child and maternal outcomes including time from start of IOL to delivery (source: SPR, eCRF), time in the hospital prior to and after delivery (any ward) (source: SPR, eCRF), number of calls for counselling and visits to the hospital after start of IOL including reasons for these visits and phone calls (source: eCRF). Mode of delivery; spontaneous vaginal birth, instrumental vaginal birth or caesarean section (source: SPR), time from IOL to active labour (source: SPR), primary method of IOL (source: SPR), additional methods of cervical ripening prior to amniotomy (source: SPR, eCRF), duration of stay at hospital after delivery (source: SPR), unscheduled visits postpartum (source: SPR, patient chart, the National Patient Register), readmission postpartum within the first month (source: SPR, patient chart, the National Patient Register).

#### Future pregnancy outcomes

The number of future deliveries, start and mode of delivery, fear of childbirth and patient satisfaction with future births will be followed through the SPR 10 years after delivery.

### Power calculation

Non-inferiority will be tested with a two-sided 95% CI and 80% power. Calculating with 2.8% (based on data from the SPR 2014–2018^5^) for the primary composite outcome in outpatients and 2.3% in inpatients and a 5% drop-out rate, 8891 women need to be randomised to achieve a probability ≥0.80 that the upper limit of a two-sided 95.7% CI for the difference in primary outcome will be less than the non-inferiority margin 1.5%. The vaginal delivery rate was 88% in a group of women fulfilling the eligibility criteria (SPR, 2014–2018^5^). Assuming a vaginal delivery rate of 90% in the outpatient arm, calculating with 80% power, a two-sided 99.3% CI, a non-inferiority margin of 1.5% and a 5% drop-out rate, 2119 women need to be randomised to each arm induced with either balloon catheter or oral misoprostol to assess efficacy of either method in the different IOL settings.

### Study monitoring and safety

The study is monitored by a professional monitor and a data safety monitoring board (DSMB) including experts in the fields of obstetrics, midwifery, paediatrics and statistics. The DSMB is independent from the sponsor and steering group, and DSMB members have no competing interests. The roles of the monitor and DSMB are defined in the study protocol, monitor contract and DSMB charter.

The monitor ensures that the study is carried out according to the protocol and that data are collected, documented and reported according to International Council for Harmonisation – Good Clinical Practice (ICH-GCP) and applicable ethical and regulatory requirements. Study sites are/will be monitored before the start of recruitment, during the study and after the study as specified in the study’s monitoring plan.

Principally, all components of the primary safety outcome classify as adverse events (AE) or serious adverse events (SAE). As the coordinating principal investigator and the steering group should remain blinded throughout the study, the responsibility for collecting, follow-up, classification and reporting of AE, SAE and suspected unexpected serious adverse reactions (SUSAR) has been delegated to the DSMB in accordance with the Swedish Ethical Review Authority, Swedish Medical Products Agency as well as European Medicines Agency approval. AE, SAE and SUSAR are reported by study staff directly in the eCRF. The DSMB will inform the sponsor in case they judge that reported AE, SAE or SUSAR affect the safety of the study participants. The sponsor representative, steering group and DSMB have at least one meeting every 6 months.

Three interim safety analyses have been prespecified. The first safety analysis after the first 1000 randomised women had given birth did not prompt any changes in the study protocol. Further analyses are planned after 3500 and 6000 randomised women have given birth. Patient groups that are studied in subanalyses during the safety analyses are primiparous and multiparous women, women induced due to PROM or other indications, and women induced primarily with balloon catheter or oral misoprostol. Results are only available for the DSMB; whereas, the sponsor, steering group and statistician remain blinded. According to the DSMB charter, due to the non-inferiority design, the study should not be stopped due to a positive difference in the primary composite endpoint and neither for futility (conditional power). The main aim is to assess safety for the women and foetuses/children involved in the study. If deemed necessary, the DSMB will make recommendations to the sponsor and steering group regarding continuation, modification or termination of any or all arms of the study. All changes to the study protocol and conduction of the study must be approved by the Swedish Ethical Review Authority, the Swedish Medical Products Agency (medical technical device) and the European Medicines Agency (investigational medical product).

Study participants are covered by the Swedish Patient Injury Act and the Pharmaceutical Insurance provided by the healthcare regions.

### Data collection, management, and analysis

#### Data collection

Data will be collected from Swedish quality and mandatory health registers, the study eCRF and questionnaires. Data from the different sources will be linked through the Swedish personal identification number before being replaced by a study ID. Register data will be collected from SPR,^26^ the Swedish Neonatal Quality Register (SNQ),^44^ the Swedish Ambulance Register,^45^ registers at the National Board of Health and Welfare (National Patient Register, the Cause of Death Register, the Prescribed Drug Register)^46^ and Statistics Sweden (SCB),^47^ see table 2. All registers have close to full coverage due to electronic data transfer from hospital charts.

For data collection in the questionnaire and interview part of the study, please see above.

#### Data management

Study-related information will be stored securely in locked file cabinets in areas with limited access at each study site as well as in the study’s eCRF. The eCRF is secured with the hospital staffs’ electronic identification card and a 2-step password-protected access system, and care providers at a certain study site only have access to data from their own site. All electronic data are handled according to the General Data Protection Regulation (GDPR) during collection, analysis and reporting.

#### Data analyses

The final statistical analysis plan (SAP) will be set prior to data lock. All main analyses will be performed according to ITT, and complementary analyses will be performed on the per-protocol population. The statistician performing the analyses will be blinded to allocation and primary IOL method.

For comparison between the two allocation groups, Fisher’s exact test will be used for dichotomous variables, Fisher’s non-parametric permutation test for continuous variables, Mantel-Haenszel χ² test for ordered categorical variables and Pearson’s χ² test for non-ordered categorical variables. For all comparisons between the two groups, regarding dichotomous and continuous variables, mean differences with 95% CI will be calculated.

For the primary outcome variable and other dichotomous outcome variables, multivariable binary regression will be used for the adjustment. For continuous variables, analysis of covariance (ANCOVA) will be used for the adjustment.

For dichotomous variables, risk difference with 95% CI and risk ratio with 95% CI will be calculated between the two groups and 95% CIs for the estimated proportions. The distribution of continuous variables as well as change in continuous variables will be given as mean, SD, median, minimum, maximum, and first and third quartiles. Categorical variables will be given as number and proportion. Exploratory analyses will be performed on subgroups stratified for parity (primiparous vs multiparous women), initial method of IOL (balloon catheter vs oral misoprostol), Bishop score in women without PROM (<3 versus ≥3), indication for IOL (PROM vs other than PROM) and reported travel time to the hospital (<30 min and ≥30 min).

For the health economy analysis, the primary analysis of effectiveness will be comparable to the composite outcome defined as primary outcome.^48^ If non-inferiority is established in the primary analyses, a simpler cost-minimisation analysis will be conducted, that is, only analysing differences in economic costs (and not in relation to the clinical outcomes). All analyses will focus on differences in means between costs and between clinical outcomes. Since cost data are typically non-normal, sampling uncertainty on differences in costs and cost-effectiveness will be assessed by non-parametric bootstrapping.^48^ Applying a simulation model, cost effectiveness for a longer time horizon will be assessed by extrapolation. To do so, associations between neonatal and maternal morbidity and health outcomes later in life will be estimated based on available epidemiologic literature.

For the qualitative studies, data analysis will be conducted by either phenomenology with a lifeworld approach^49^ or content analysis.^43 50^

### Project organization

The study has been planned and is organised by the OPTION steering group including the authors for this publication apart from HS who is a PhD student in the project. Region Västra Götaland, a public healthcare region in western Sweden, is sponsor of the OPTION trial with first author VS being coordinating principal investigator and sponsor representative. Gothia Forum, the research support unit for Region Västra Götaland, serves with legal aspects of the study.^51^ The study is supported by the Swedish Network for National Clinical Studies in Obstetrics and Gynecology (SNAKS).^52^ Omda Health Analytics provides the platform for both SPR and SNQ and built the eCRF and randomisation module.

The OPTION study group including all principal investigators as well as relevant study staff at the different study sites has monthly meetings to plan and discuss study progress, receive feedback from the monitor and ensure adherence to the study protocol and participant safety. The study is monitored by a monitor and DSMB as described above.

### Patient and public involvement statement

Patient representatives have been involved in planning of the study, regularly join DSMB meetings and will be involved in the further steps such as discussion of the SAP and dissemination of results.

## Ethics and dissemination

The Swedish Ethical Review Authority approved the study (3 June 2020; 2020-02675 with amendments 2021-03045, 2022-00865-02, 2023-01252-02, 2024-00560-02, 2024-2024-04597-02). The Swedish Medical Products Agency approved the study for the medication arm (25 August 2020, EudraCT number: 2020-000233-41; 5.1-2020-60240 with amendments 5.1-2022-73500, 5.1-2023-630). Due to changed regulation, in 2023, the study medication arm was transferred and approved by the European Medicines Agency (23 October 2023, EU CT Number: 2023-507164-39-00; CTIS 5.1.2-2023-099775 with amendments 5.1.2-2024-081916, 5.1.2-2025-036291). The Swedish Medical Products Agency approved the study for the medical device arm (6 April 2021, CIV-ID: CIV-20-09-034712; 5.1-2021-14812 with amendments 5.1-2022-14252, 5.1-2023-596, 5.1-2024-8886, 5.1-2024-55554). The medical device arm was transferred to Regulation (EU) 2017/745 (23 December 2024, 5.1-2025-24242 and amendment 5.1-2025-6050).

The current version number of the study protocol is 14, dated 10 June 2025, and finally approved 8 July 2025.

Pregnant women and participants are informed on the study and results from the OPTION trial through the study homepage and Instagram account, articles in public media, contributions to different pods, radio and television programmes and webinars organised by the Swedish Network for National Clinical Studies in Obstetrics and Gynecology (SNAKS).

The study will involve more than 80% of all maternity hospitals in Sweden, which will allow for a smooth implementation of any new routine after the study’s conclusion.

Results from the study will be published in peer-reviewed open access scientific journals and presented on scientific conferences as well as on the study homepage^27^ and in public and social media. Data from the trial might become part of future meta‐analysis.

Authorship for publications derived from this trial will comply with criteria for authorship as defined by the International Committee of Medical Journal Editors (ICMJE).

## Discussion

During spontaneous labour, women typically stay at home during cervical ripening and are admitted to the hospital when active labour begins.^2^ Small RCTs suggest that outpatient IOL is well-tolerated,^53^ could enhance patient satisfaction^13 14 17 18^ and reduce healthcare costs,^22^ but these studies lack sufficient power to assess safety outcomes. Both a 2020 Cochrane review^12^ and a recent review^11^ concluded that current evidence on the safety and efficacy of outpatient IOL is insufficient, making this study crucial before the method is widely implemented.

The strengths of this trial are several: It is a national multicentre clinical R-RCT engaging almost all delivery units. It is endorsed by the Swedish Network for National Clinical Studies in Obstetrics and Gynecology (SNAKS),^52^ a network that has successfully facilitated multiple clinical trials previously. The study is run as an R-RCT with randomisation and data collection based on high-quality Swedish healthcare and quality registers. The registries with almost 100% coverage will provide nearly complete follow-up even of long-term consequences such as future pregnancy outcomes. Another strength is that the study assesses experience and satisfaction of patients, partners and care providers, which will provide valuable insights when considering implementation of outpatient IOL. Health economic analyses will be central in determining whether outpatient IOL can become clinical routine.

The low prevalence of serious adverse outcomes in low-risk pregnancies, especially in high-resource settings, challenges the feasibility of RCTs on outpatient IOL. The opportunity for an RCT on outpatient IOL in Sweden occurred after the introduction of new guidelines offering all women at 41+0 gestational weeks IOL. In all deliveries, IOL rates have increased with approximately 38% without the allocation of extra resources. There is significant national interest in participating in the OPTION trial, demonstrated by 31 out of 45 Swedish delivery units currently recruiting and additional 6 units planning for participation.

In summary, this national prospective multicentre register-based randomised controlled trial aims to assess the safety and efficacy of outpatient IOL vs hospital IOL in low-risk IOLs. Primary endpoints include neonatal safety, defined by a composite outcome, and efficacy, measured by the proportion of vaginal deliveries. Due to increasing induction rates, outpatient IOL is rapidly gaining popularity worldwide. This study will provide a comprehensive approach and evaluation of outpatient IOL encompassing all relevant aspects including safety for both mother and child, experience of pregnant women, partners and care providers, as well as economic implications.

## Supplementary material

10.1136/bmjopen-2024-093972 online supplemental file 1

10.1136/bmjopen-2024-093972 online supplemental file 3
