# Supplementary material for: The OPTION trial: outpatient induction of labour – study protocol for a prospective, non-inferiority, multicentre randomised controlled trial
Source: BMJ Open. 2025 Aug 13;15(8):e093972. doi: 10.1136/bmjopen-2024-093972 (PMC12352190; doi:10.1136/bmjopen-2024-093972)
Supplement: online supplemental file 1 [file bmjopen-15-8-s001.docx]

**Supplemental Table 1** Single ballon catheters approved for induction of labour in the OPTION trial

| **Manufacturer** | **Model** | **Date of approval** |
| --- | --- | --- |
| Coloplast | 1. Coloplast X-FLOW® Prostatectomy short catheter straight tip 3-way 30-50 ml silicone CH FR 22 (Coloplast catheter) | Study start |
| BD AB (Becton, Dickinson and Company) | 1. BARD Biocath 14Ch/Fr (4,7 mm), 10 ml. REF: 226514 2. BARD Biocath 18Ch/Fr (6,0 mm), 10 ml. REF: 226518 3. BARD Biocath 20Ch/Fr (6,7mm), 30 ml. REF: 226620 4. BARD Biocath 22Ch/Fr (7,3 mm), 30 ml, REF 226622 5. BARD Biocath 24 Ch/Fr (8,0 mm), 10 ml REF: 226524 6. BARD Hematurikateter, förstärkt, tvåvägs, 18Ch/Fr (6,0mm), 30/50 ml. REF: 1852H18 7. BARD Hematurikateter Nylon spiral 3 vägs, 20 Ch/Fr (6,7mm), 30/50 ml. REF: 2551H20CE 8. BARD Hematurikateter förstärkt, tvåvägs, 22 Ch/Fr (7,3mm) 30/50 ml REF 1852H22 9. BARD Hematurikateter, förstärkt, två vägs, 24Ch/Fr (8,0mm) 30/50 ml. REF: 1857H24 10. BARD Bardia 18 Ch/Fr (6,0mm) 35 ml REF: 123618CE 11. BARD Bardia 22 Ch/Fr (7,3mm) 30 ml REF: 123422CE 12. BARD Bardia 24 Ch/Fr REF 123624CE | March 14^th^ 2025 |
| Teleflex Incorporated | 1. RÜSCH Gold 22 CH (7,3mm) 30/50 ml REF 180630 2. RÜSCH GOLD 3 way haematuria 24 Ch.Fr (8,0mm) 30-50 ml. REF: 183960 3. RÜSCH Softsimplastic 24 Ch (8,0mm) 30 ml. CAT No 664130 | March 14^th^ 2025 |
