# Supplementary material for: The OPTION trial: outpatient induction of labour – study protocol for a prospective, non-inferiority, multicentre randomised controlled trial
Source: BMJ Open. 2025 Aug 13;15(8):e093972. doi: 10.1136/bmjopen-2024-093972 (PMC12352190; doi:10.1136/bmjopen-2024-093972)
Supplement: online supplemental file 3 [file bmjopen-15-8-s002.pdf]

OPTION – OutPatienT InductiON

Study into induction of labour *at home* compared to *in hospital*,

a randomised controlled study

Information to trial subjects

We would like to ask if you wish to participate in a research project. In this document, you will find information about the project and what it means to participate.

What kind of project is it, and why do you wish for me to take part?

This research study aims to compare the induction of labour at home with induction in hospital. In Sweden, between one in three and one in four deliveries are induced. There is usually a medical reason for inducing labour, such as the pregnancy being overdue, illness on the part of the woman or baby, or the amniotic fluid (waters) having broken without labour having started. Today, induction is often done by taking the drug misoprostol by mouth or by inserting a balloon catheter into the uterus via the cervix. Both methods are well studied and safe and aim to soften and open the cervix. This phase of induction is equivalent to the ripening phase of the cervix at the start of spontaneous labour.

Which of the methods is chosen to induce labour will depend on how ripe the cervix is, whether you have previously given birth and whether the waters have broken. You and your doctor will decide together which method is appropriate.

In many other countries, including Denmark and Finland, labour is induced in hospital and women are then offered the opportunity to go home and remain at home during the ripening phase. However, to date no studies have been performed in a scientifically satisfactory manner into whether it is as safe and effective to remain at home during this phase. Equally, there is a lack of knowledge of the effects of induction on the woman's quality of life, self-confidence, mood, experience of childbirth and breastfeeding. In this study, we will examine whether inducing labour at home is as safe and effective as being in hospital, and whether and how it affects the woman. The knowledge gained may increase the opportunities to provide individualised treatment and to improve the care of women where labour needs to be induced.

The study is being conducted at a number of hospitals in Sweden. The research principal for the project is Västra Götalandsregionen, Sahlgrenska Universitetssjukhus, Göteborg. The study has been approved by the Swedish Ethical Review Authority and the Swedish Medical Products Agency.

How is the study conducted?

If you consent to take part in the study, we will begin by assessing whether you and your foetus meet the criteria for participation. We will check your blood pressure, urine sample and state of health. The foetus will be examined using CTG (cardiotocography, which records the

baby's heart sounds) and an abdominal ultrasound in order to measure the volume of amniotic fluid and to check that the baby is lying with its head down. Your cervix will be examined to assess how ripe it is. Labour will then be induced using the method agreed between you and your doctor. You will remain at the hospital for at least 45 minutes afterwards in order to check that you are feeling well and that your labour pain does not start rapidly. If the insertion of a balloon catheter has been chosen, CTG will also be performed following insertion. During this time, you will complete Questionnaire I, where you estimate your self-confidence, quality of life, thoughts about pain, sense of coherence and feelings of depression (this will take approx. 15 minutes). The questionnaire will be sent to your e-mail address and/or via SMS to your mobile phone. You will then be randomly drawn to either: (i) home induction, i.e. where you are at home (or at a patient hotel) during the ripening phase (Group 1), or (ii) hospital induction, i.e. where you remain in hospital during the ripening phase (current standard care) (Group 2).

Group 1: Home induction

- If your labour has been induced using Angusta® tablets (misoprostol), you will continue taking tablets at home at intervals of not less than two hours (up to a maximum of eight tablets over 24 hours). You will be given the tablets to take home, along with a precise schedule showing when you must take them.
- If your labour has been induced using a balloon catheter, it can be left in place until it falls out of its own accord, though for no more than 12 or 24 hours (depending on the model). You can move around freely, sleep and eat as normal at home. We advise that you do not have intercourse or take a bath if you have a balloon catheter, but it is fine to take a shower.

You will be given clear information to take home with you about when you need to call the hospital, with access to advice over the telephone from a midwife. Once the onset of labour begins or your waters start to break – or, if they have already broken, they change colour – you should contact the hospital for an assessment. Contact the Maternity Unit immediately if you are experiencing persistent labour pains, abdominal pain or bleeding.
If labour has not started following treatment with eight doses of Angusta® tablets, or if the balloon catheter has not fallen out after 12 or 24 hours (depending on the model), you will be given a follow-up appointment for a fresh examination and a decision will be made on how to continue with the induction. If you have not been induced because your waters have broken and your cervix remains unripe, you may remain at home for a further 24 hours, either using the same method or after switching method. On the third day following the start of induction you will return to the hospital, and all treatment from then on will take place at the hospital. If you have been induced because your waters have broken, you will be admitted to hospital from the second day of induction.

Group 2: Hospital induction

- If your labour has been induced using Angusta® (misoprostol), you will continue taking tablets at intervals of not less than two hours (up to a maximum of eight tablets over 24 hours).

- If your labour has been induced using a balloon catheter, it can be left in place until it falls out of its own accord, though for no more than 12 or 24 hours (depending on the model). You can move around freely, sleep and eat. We advise that you do not have intercourse or take a bath if you have a balloon catheter, but it is fine to take a shower.

If you are experiencing persistent labour pains, abdominal pain or bleeding, you will be assessed on site by a midwife and/or doctor. If labour has not started following treatment with the maximum of eight doses of Angusta® tablets, or if the balloon catheter has not fallen out after 12 or 24 hours (depending on the model), a new assessment will be made of how to continue with the induction. If your cervix remains unripe, induction will continue at the hospital, either using the same method or after switching to the other method. You will remain at the hospital for the entire induction.

Follow-up for all participating women

Approximately 12 weeks after the birth, you will receive Questionnaire II by e-mail and/or SMS. This contains the same questions that you answered on Questionnaire I – in other words: estimate of your self-confidence, quality of life, thoughts about pain, sense of coherence and feelings of depression – as well as two additional metrics where you appraise your experience of childbirth and your confidence in breastfeeding 12 weeks after the birth (this will take approx. 40 minutes). This questionnaire also contains questions where you can share, in your own words, your experience of the induction and the delivery (the time taken for this part depends on how much you wish to write about your experiences).

Possible consequences and risks in taking part in the study

Your participation is voluntary, and you are fully entitled to decline to take part in the study without further explanation and without this affectingr your current or future care. On the other hand, you cannot be offered further induction at home if you decide to end your participation in the study. If you wish to end your participation, you must initially contact the Maternity Coordinator/midwife at the Maternity Unit, followed by one of the people responsible for the study (see below).

For those women being induced in hospital, participation means receiving the method that is standard today. There is a small risk of severe labour pain following the use of both the balloon catheter and misoprostol tablets. If this should occur in those women who are at home, they will contact the Maternity Coordinator/midwife by telephone. Women at home do not have the same opportunity to receive immediate attention as those in hospital. For this reason, only women at low risk of complications during the ripening phase may take part in the study.

Many women can find it tedious and stressful being in hospital during induction. Those women who are randomly drawn to receive induction at home then have a greater opportunity to move around freely and live as normal, taking showers and baths, eating, resting and sleeping – in the same manner as women whose labour starts of its own accord.

What will happen with my details?

As well as the questionnaires that you will complete, we will collect details about you, your delivery and your baby from your medical record and specific questions, e.g. how long it takes for you to get to the Maternity Department, from the Pregnancy Register, the Swedish Neonatal Quality Register, the Patient Register, the Register of Causes of Death, the Prescribed Drug Register, the Swedish Ambulance Register and from Statistics Sweden (Population Register, Education Register and Income & Taxation Register). The study personnel may also have occasion to go into your National Patient Summary (NPÖ) in order to see whether you have received any treatment that may be related to your participation in the study at a unit other than the Women's Clinic where you have been included in the study. On the study you will be given a code with which your completed questionnaires, the administration details about the induction of your labour, your patient record and register data will be marked. The list that shows your code is stored separately from the database and is stored locked away with the person in charge of the study project. Only researchers involved in the study have access to the database, and all documents are stored for at least 25 years in accordance with the Swedish Data Act. In the OPTION study, the data and code key will be stored for at least 25 years in order to facilitate long-term monitoring of women and children via the registers. An independent monitor, representatives of the Swedish Medical Products Agency or other relevant authorities may check the data that has been recorded in order to confirm that the study is being conducted correctly. They can then compare the information from the research forms with those in your patient record. These people are also covered by a duty of confidentiality. Your data is confidential, and your answers and results will be processed in such a way as to prevent unauthorised access. When the data from the study is published, it will not be possible to identify individuals.

Responsibility for your personal data lies with the Sahlgrenska University Hospital, Gothenburg. According to the EU General Data Protection Regulation (GDPR) (EU 2016/679), you have the right to access the data about you that is processed in the study free of charge, and, where appropriate, to have your data corrected. You can also request that data about you/your child be erased and that the processing of your personal data be restricted. If you wish to access the data, you can contact any of the researchers in charge. If necessary, the Data Protection Officer can also assist you with this. You can contact the Data Protection Officer at: Sahlgrenska University Hospital, Data Protection Officer, SE-413 45 Gothenburg, telephone [redacted]. If you are unhappy with how your personal data is being processed, you have the right to file a complaint with the supervisory Swedish Authority for Privacy Protection's (IMY).

How do I find out information about the results of the study?
The results of the study will be presented in the EU database for clinical trials "Clinical Trials Information System – CTIS" and published on the study's website, reported in scientific articles, presented at scientific conferences and be included as part of theses and dissertations. If you would like to find out the results of the study, you may contact the researchers in charge. See contact details above.

Insurance, compensation

The Swedish Patient Injury Insurance scheme applies. No special compensation will be paid for taking part in the study.

**Doctor in charge of research**: [redacted]	Local researcher in charge at Women's Clinic [redacted]

OPTION – OutPatienT InductiON Study into induction of labour *at home* compared to induction of labour *in hospital* – a randomised controlled study.

Consent to take part in the study by the pregnant woman

I have been verbally informed of the above study and have read the attached information. I have been given the opportunity to ask questions and have had any questions answered. I am aware that my participation is completely voluntary. I am aware that I may end my participation at any time and without further explanation, and that this will not affect my current or future treatment (other than induction taking place in hospital in accordance with standard treatment).

With my signature, I consent to:

- Participating in this study
- My and my baby's study data (personal data) being processed as described, and data collected about me and my baby being kept and processed electronically by those responsible for the study
- Information about me and my baby being obtained from our national quality and health registers and from Statistics Sweden for use in the research described
- An independent monitor, representatives of the Swedish Medical Products Agency or other relevant authorities checking the data that has been recorded in order to confirm that the study is being conducted correctly
- My study data and that of my baby being linked to my partner's data if my partner opts to take part in the study. Partner's personal ID number: ____________________

Date, entered by trial subject ____

Signature of trial subject ____

Personal ID number of trial subject ____

Printed name of trial subject ____

Personal ID number of other parent____

Telephone/Mobile No.____

E-mail____

The undersigned researcher/health care professional has gone through and explained the purpose of the study to the above trial subject and has obtained the trial subject's consent. The trial subject has been given the information to participants.

Date, signature____

Print name and job title____
