# Supplementary material for: The OPTION trial: outpatient induction of labour – study protocol for a prospective, non-inferiority, multicentre randomised controlled trial
Source: BMJ Open. 2025 Aug 13;15(8):e093972. doi: 10.1136/bmjopen-2024-093972 (PMC12352190; doi:10.1136/bmjopen-2024-093972)
Supplement: Supplementary file 3 [file bmjopen-2024-093972.reviewer_comments.pdf]

PEER REVIEW HISTORY

BMJ Open publishes all reviews undertaken for accepted manuscripts. Reviewers are asked to complete a checklist review form and are provided with free text boxes to elaborate on their assessment. These free text comments are reproduced below.

ARTICLE DETAILS

Title (Provisional)

THE OPTION TRIAL: Outpatient induction of labour. Study protocol for a non-inferiority, multicentre randomised controlled trial

Authors

Sengpiel, Verena; Sangskär, Heléne; Wennerholm, Ulla-Britt; Elden, Helen; Gemzell-Danielsson, Kristina; Graner, Sofie; Wallström, Tove; Hesselman, Susanne; Domellöf, Magnus; Jonsson, Maria; Brismar Wendel, Sophia; Herbst , Andreas; Kopp-Kallner, Helena; Carlsson, Ylva

---

VERSION 1 - REVIEW

Reviewer 1

Name Baev, Oleg

Affiliation I M Sechenov First Moscow State Medical University, Obstetrics, gynecology

Date 02-Nov-2024

COI I have a joint publication with Wennerholm, Ulla-Britt.

The jointly published study was multicenter and I had no personal contact with Ulla-Britt.

Dear authors, I find your protocol quite detailed and well thought out. But some points need clarification, which should be indicated in the text.

Since the study is devoted to assessing safety, you should specifically indicate what you mean by «low risk IOL». And also give a definition for a clear understanding of the term «prolonged latent phase» and «accelerated fetal growth»

Reviewer 2

Name Nicholson, Sarah

Affiliation Royal College of Surgeons in Ireland

**Date**	**22-Nov-2024**
**COI**	**None**

Comments and questions

This is a worthwhile and much-needed study in the face of increasing induction rates worldwide. The plan is for a large sample size which may help assess safety outcomes taking into account the rarer events that occur within the cohort. Thank you for the opportunity to review. I have some minor comments and questions for clarification:

1. Line 177 – is there an upper limit on the age group included?
2. Line 207 – is there a CTG performed on all women before IOL occurs? In the text it appears that only those with a balloon catheter receives a CTG, whereas one might expect that may be more appropriate for every woman, but particularly those receiving hormonal induction agents?
3. How are women counselled to present if hyperstimulation occurs?
4. Table 1 - Are those with PROM routinely tested at the time of PROM for the presence of Group B streptococcus? Are women required to take their temperature at home if IOL is for PROM?
5. Is this study also powered to assess the differences within nulliparous and multiparous groups as the result of ripening is likely to differ between the two, with nullips being the more difficult group to induce?
6. Line 448 - what small RCTs - reference?
7. Is there a set protocol being followed when the patient is readmitted or do local protocols differ regarding oxytocin use etc?
8. Given that women are up to 60 mins from the hospital, is there a fallback system in place for those who experience precipitous labour during this process?
9. I note many references appear to be from some time ago – is the literature review up to date?

I look forward to seeing the results of this largescale RCT.

**Reviewer**	**3**
**Name**	**Rengaraj, Sasirekha**

**Affiliation**	**JIPMER**
**Date**	**14-Feb-2025**
**COI**	**None**

Huge Multi centric non-inferiority trial; Well designed and written protocol

VERSION 1 - AUTHOR RESPONSE

Reviewer: 1
Dr. Oleg Baev, I M Sechenov First Moscow State Medical University, FSBI National Medical Research Center for Obstetrics Gynecology and Perinatology named after Academician V I Kulakov
Comments to the Author:
Dear authors, I find your protocol quite detailed and well thought out. But some points need clarification, which should be indicated in the text.

Thank you for taking your time to review our paper.

Since the study is devoted to assessing safety, you should specifically indicate what you mean by «low risk IOL».

Thank you for pointing out the need for clarification. We now have defined the term low-risk IOL in the section "Recruitment, screening and inclusion", page 8, line 242: "Women with a term, singleton, live foetus in stable cephalic presentation, unripe cervix, normal CTG prior to IOL, and an anticipated uncomplicated cervical ripening phase—i.e., not requiring maternal or foetal monitoring—are defined as low-risk IOL and eligible for inclusion. Detailed in- and exclusion criteria are presented in Table 1.“

And also give a definition for a clear understanding of the term «prolonged latent phase» and «accelerated fetal growth»

The term "prolonged latent phase" has not been defined by the study protocol and refers to the hospital's clinical routine for assigning the diagnosis. Usually, it is defined as more than 18 hours of painful contractions without cervical dilatation reaching 5 cm. As this condition is not specified by the study protocol and may vary between study sites, we would prefer to not further describe or define it in the study protocol.

There is no national consensus for "accelerated fetal growth" depending on time

between measurements as well as delta in weight measurements but usually a delta of 10-15% weight increase with 2-4 weeks between measurements is regarded as suspected growth acceleration. We have added the following text to table 1: “Small for gestational age (SGA) defined as <2 and in case of diabetes either large for gestational age (LGA) defined as >2 standard deviation according to Marsal et al (54) or suspected growth acceleration (according to the hospital’s guidelines).”

Reviewer: 2
Dr. Sarah Nicholson, Royal College of Surgeons in Ireland
Comments to the Author:
See attached file.

Comments and questions

This is a worthwhile and much-needed study in the face of increasing induction rates worldwide. The plan is for a large sample size which may help assess safety outcomes taking into account the rarer events that occur within the cohort. Thank you for the opportunity to review.
Thank you for your thorough review of this manuscript. Please find our comments below.

I have some minor comments and questions for clarification:

1. Line 177 – is there an upper limit on the age group included?
   Yes, the upper age limited is 45 as indicated under inclusion criteria. We added the information on page 8 line 232: “Women aged 18 to 45 years planned for IOL between 37+0 and 41+6 gestational week will be screened for eligibility (Table 1).”

2. Line 207 – is there a CTG performed on all women before IOL occurs? In the text it appears that only those with a balloon catheter receives a CTG, whereas one might expect that may be more appropriate for every woman, but particularly those receiving hormonal induction agents?
   A CTG is performed for all women before inclusion which occurs immediately before induction. A new CTG is performed immediately after inserting a balloon catheter but not for women receiving the first tablet of misoprostol. In this group a CTG will be performed only if indicated, e.g. start of contractions.

   We have tried to clarify the procedures, please see page 8 line 241: “Screening for eligibility takes place at the hospital visit for start of IOL

including medical history, blood pressure, vaginal examination, abdominal ultrasound, and CTG. Women with a term, singleton, live foetus in stable cephalic presentation, unripe cervix, normal CTG prior to IOL, and an anticipated uncomplicated cervical ripening phase—i.e., not requiring maternal or foetal monitoring—are defined as low-risk IOL and eligible for inclusion."

And page 9 line 276: "For women who received a balloon catheter a CTG is registered after insertion, before randomisation, see Figure 2. For women who received the first dose of oral misoprostol an additional CTG is only registered if indicated, e.g. start of contractions."

3. How are women counselled to present if hyperstimulation occurs?

   We have tried to further specify how women in the outpatient group are counselled, please see page 10 line 309: "Women randomised to outpatient IOL, receive detailed oral and written instructions to contact the hospital (and refrain from further intake of oral misoprostol) at start of contractions, in case of PROM (if PROM was not the reason for IOL), when the balloon catheter is expelled, sudden change/decrease in foetal movements, vaginal bleeding, abdominal pain, fever and/or feeling unwell, and in case of IOL due to PROM: any change in amniotic fluid colour. These women are counselled to come to the hospital for a clinical evaluation. Women are also encouraged to call in case they feel unsure about anything."

   Please see even answer to question 8 below.

4. Table 1 - Are those with PROM routinely tested at the time of PROM for the presence of Group B streptococcus? Are women required to take their temperature at home if IOL is for PROM?
   The testing strategies for PROM patients are not part of the study protocol and all hospitals continue with their clinical routine. Recommended clinical routine in Sweden is to not test for GBS in case of PROM or start of labour but to administer prophylactic intravenous antibiotics in active labour for the following groups:

   - Child from a previous pregnancy affected by neonatal GBS infection
   - GBS detected in urine (regardless of bacterial count) during the current pregnancy
   - Preterm birth (<37 completed weeks of gestation)
   - Prolonged rupture of membranes (≥18 hours)

- Intrapartum fever (≥38 °C)

Women with PROM are advised to take their temperature twice daily. We have added the reference to the Swedish routines regarding the risk-based (rather than screening-based) GBS management to the manuscript, please see Table 1: “Known colonisation with group B streptococci or previous pregnancy complication linked to group B streptococci (55)”.

5. Is this study also powered to assess the differences within nulliparous and multiparous groups as the result of ripening is likely to differ between the two, with nullips being the more difficult group to induce?
   No, the trial is powered to study the primary safety outcome in the whole group of all 8891 study participants. Parity is one of the variables randomisation is stratified on and sub analyses for nulliparous and multiparous are planned. Depending on the ratio of included nulliparous and multiparous, the trial might be powered to study some of the other outcomes, e.g. the efficacy outcome, in nulliparous and multiparous women separately.

6. Line 448 - what small RCTs - reference?
   Thank you for pointing out the lack of references provided. We have added the references as suggested, please see page 17 line 560: ” Small RCTs suggest that outpatient induction of labour (IOL) is well-tolerated (52), could enhance patient satisfaction (13, 14, 17, 18) and reduce healthcare costs (22), but these studies lack sufficient power to assess safety outcomes.”

7. Is there a set protocol being followed when the patient is readmitted or do local protocols differ regarding oxytocin use etc?

   The study protocol specifies patient care for the first two days of cervical ripening. In case of admission for completed cervical ripening, a complication, or the third day of induction, patient care follows local guidelines. Routinely, when the cervix is ripe, membranes are artificially ruptured (not in case of PROM), and an oxytocin infusion is initiated within 2 hours if regular contractions are not present, according to the guidelines from the Swedish Society of Obstetrics and Gynecology.

   We have added the information and reference, please see page 8 line 301: “Thereafter, all participants are treated according to clinical routine at their hospital. Usually, IOL will proceed with oxytocin infusion in case of PROM and amniotomy and subsequent oxytocin infusion in women with intact membranes (29).”

8. Given that women are up to 60 mins from the hospital, is there a fallback system in place for those who experience precipitous labour during this process?
   There is no specific fallback system other than for all women awaiting spontaneous start of labour. If needed, women can call an ambulance for fast and safe transportation to the hospital. Data from the Swedish ambulance register will be collected to establish how often women had to be transported from home by ambulance.

   However, in- and exclusion criteria, patient information, and screening exams specified in the study protocol have been designed to prevent precipitous labour at home as far as possible:
   - Women with previous precipitous labour are not eligible.
   - Women with Bishop score ≥6 (ripe cervix) are not eligible.
   - All women stay for a 45 min observation time at the hospital after start of induction and before randomization.
   - Women are carefully advised to contact the hospital in case something starts to feel different, and they are advised to come back to the hospital in case of start of labour. The advice given to trial participants thus differ from advice given to women calling for start of spontaneous labour.

9. I note many references appear to be from some time ago – is the literature review up to date?
   The literature referred to is based on a systematic review by Sangskar et al that was performed when planning the OPTION trial. An updated literature review did not reveal any new RCTs powered to study efficacy or safety of outpatient IOL.
   However, we included the recently published studies on patient and partner experience, please see page 7 line 193: "Studies have indicated that the home environment contributes to physical and emotional comfort and might enhance women's birthing experiences (13-18)."

   Page 7, line 196: "Moreover, the partner's experience has hardly been studied (19), despite the partner often being the main support person during outpatient IOL. Only a handful of studies have investigated health care provider experience, yielding both positive feedback and certain concerns (20, 21)."

   Page 17 line 560: "Small RCTs suggest that outpatient induction of labour (IOL) is well-tolerated (52), could enhance patient satisfaction (13, 14, 17, 18), and reduce healthcare costs (22), but these studies lack sufficient power to assess safety outcomes."

I look forward to seeing the results of this largescale RCT.

Reviewer: 3
Dr. Sasirekha Rengaraj, JIPMER
Comments to the Author:
Huge Multi centric non-inferiority trial; Well designed and written protocol

Thank you for taking the time to review our paper.
